# No sex-related differences in CGI-S score reductions in adult patients with acutely exacerbated schizophrenia treated with Risperidone ISM

**DOI:** 10.1192/j.eurpsy.2025.767

**Published:** 2025-08-26

**Authors:** C. Sherifi, J. Martínez González, L. Anta Carabias, M. Almendros Gimenez, C. Salazar García, C. U. Correll

**Affiliations:** 1 Medical Affairs, ROVI Biotech Ltd., Croydon, United Kingdom; 2 Medical Department, Laboratorios Farmaceuticos ROVI S.A., Madrid, Spain; 3 Department of Psychiatry and Molecular Medicine, Donald and Barbara Zucker School of Medicine at Hofstra/Northwell, Hempstead, NY; 4 Department of Psychiatry Research, The Zucker Hillside Hospital, Glen Oaks, NY, United States; 5 Department of Child and Adolescent Psychiatry, Charité Universitätsmedizin Berlin, Berlin, Germany; 6 Center for Psychiatric Neuroscience, The Feinstein Institute for Medical Research, New Hyde Park, NY, United States; 7 German Center for Mental Health, Partner Site Berlin, DZPG, Berlin, Germany

## Abstract

**Introduction:**

There is an increasing emphasis on incorporating sex differences into mental health research [Galbally *et al.* CNS Drugs 2024; 38(7):559-570; Ercis *et al.* J Affect Disord. 2024; 352:171-192]. Reports on evaluating sex-related differences in risperidone efficacy are limited [Galbally *et al.* CNS Drugs 2024; 38(7):559-570]. Recently, Risperidone ISM (Risp-ISM), a monthly long-acting injectable (LAI) formulation has been authorised in Europe, USA and some other countries.

**Objectives:**

To assess potential sex-related differences in the short-term efficacy of Risp-ISM LAI in adults with schizophrenia [Correll *et al.* NPJ Schizophr. 2020; 6(1):37].

**Methods:**

Post-hoc analysis of a double-blind (DB), randomised, placebo-controlled, 12-week study conducted in participants with acutely exacerbated schizophrenia (NCT03160521). Data from the Clinical Global Impressions-Severity (CGI-S) rating scale were analysed by sex to reveal potential differences in efficacy versus placebo. The data were analysed within three separate study groups: 75 mg Risp-ISM, 100 mg Risp-ISM and placebo using a mixed effect with repeated measures model (MMRM). Herein, CGI-S scores changes from baseline (the key secondary efficacy endpoint) are shown.

**Results:**

In the double-blind phase, 437 eligible participants were randomly assigned 1:1:1 to receive Risp-ISM 75 mg, 100 mg or placebo every 28 days. 144 (33%) were female and 293 (67%) male. Analysis showed no sex-related differences on CGI-S scores. Decreases from baseline (Figures 1 and 2) were significantly greater versus placebo at Day 8 (after first injection) and beyond in both male and female subgroups at the 100 mg Risp-ISM dose; likewise, at Day 15 and beyond for the 75 mg Risp-ISM dose.

**Image 1:**

**Figure:**
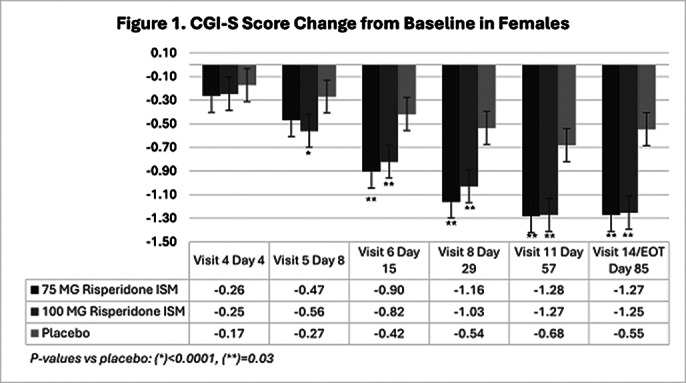

**Image 2:**

**Figure:**
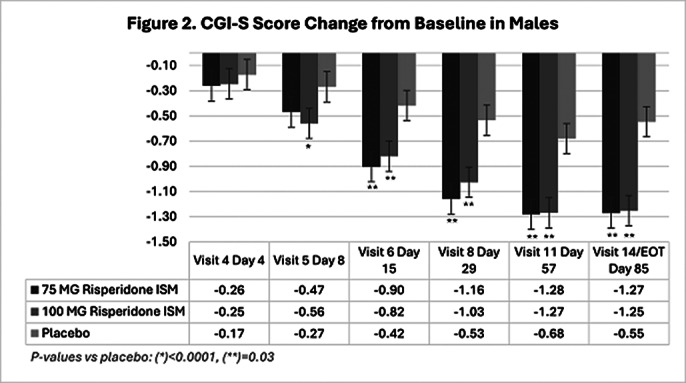

**Conclusions:**

There were no statistically significant differences in efficacy, measured as CGI-S score change from baseline, for male or female participants versus placebo with Risp-ISM 75 mg or 100 mg. Risp-ISM, using monthly intramuscular doses of 75 mg or 100 mg provided significant reduction in severity of the disease as early as 8 days after the first dose in acutely exacerbated patients with schizophrenia regardless of their sex.

**Disclosure of Interest:**

C. Sherifi Employee of: ROVI Biotech Ltd., J. Martínez González Employee of: Laboratorios Farmaceuticos ROVI S.A., L. Anta Carabias Employee of: Laboratorios Farmaceuticos ROVI S.A., M. Almendros Gimenez Employee of: Laboratorios Farmaceuticos ROVI S.A., C. Salazar García Employee of: Laboratorios Farmaceuticos ROVI S.A., C. Correll Shareolder of: Cardio Diagnostics, Kuleon Biosciences, LB Pharma, Mindpax, and Quantic., Grant / Research support from: Janssen and Takeda., Consultant of: AbbVie, Acadia, Alkermes, Allergan, Angelini, Aristo, Biogen, Boehringer-Ingelheim, Cardio Diagnostics, Cerevel, CNX Therapeutics, Compass Pathways, Darnitsa, Denovo, Gedeon Richter, Hikma, Holmusk, IntraCellular Therapies, Jamjoom Pharma, Janssen/J&J, Karuna, LB Pharma, Lundbeck, MedAvante-ProPhase, MedInCell, Merck, Mindpax, Mitsubishi Tanabe Pharma, Mylan, Neurocrine, Neurelis, Newron, Noven, Novo Nordisk, Otsuka, Pharmabrain, PPD Biotech, Recordati, Relmada, Reviva, Rovi, Sage, Seqirus, SK Life Science, Sumitomo Pharma America, Sunovion, Sun Pharma, Supernus, Takeda, Teva, Tolmar, Vertex, and Viatris.

